# Nobel Prize 2020 in Chemistry honors CRISPR: a tool for rewriting the code of life

**DOI:** 10.1007/s00424-020-02497-9

**Published:** 2020-11-26

**Authors:** Lukas Westermann, Björn Neubauer, Michael Köttgen

**Affiliations:** 1 Department of Medicine, Renal Division, Faculty of Medicine and Medical Center, University of Freiburg, Freiburg, Germany; 2 CIBSS – Centre for Integrative Biological Signalling Studies, Freiburg, Germany

Emmanuelle Charpentier and Jennifer Doudna have been awarded the 2020 Nobel Prize in Chemistry for their work on CRISPR-Cas9—a method to edit DNA. The two scientists were recognized by the Nobel Committee for their discovery that a microbial immune mechanism can be transformed into a tool that can simply and cheaply edit genomes with high precision.

In the seven decades since the discovery of the DNA double helix, technologies for analyzing, manipulating, and making DNA have enabled considerable advances in biological research [3]. But site-specific modifications of genomes remained challenging. Previous genome editing tools such as zinc-finger nucleases (ZFN) and transcription-activator–like effector nucleases (TALEN) highlighted the potential of targeted manipulation of genes, but limited targeting capacity and complicated design hampered the usability of these technologies [3]. CRISPR-Cas9 can be considered a real game changer due to its simplicity and efficiency.

CRISPR stands for *clustered regularly interspaced short palindromic repeats* and is used by bacteria to fight phage infections. In principle, the CRISPR system enables bacteria to recognize genetic sequences of invaders and target these sequences for destruction using specialized enzymes. These enzymes are called CRISPR-associated proteins (Cas) and include the DNA endonuclease Cas9 [8]. In 2011, Charpentier’s group identified a key component of the CRISPR system, an RNA molecule that is involved in recognizing foreign phage sequences in bacteria, so-called trans-activating crRNA (tracrRNA) [1]. A subsequent collaboration of the Charpentier and Doudna laboratories resulted in the landmark 2012 Science paper, which kick-started the era of CRISPR-Cas9 genome editing [6]. The paper showed that a bacterial CRISPR-Cas9 endonuclease is guided by two RNA molecules forming the tracrRNA:crRNA duplex, to direct site-specific DNA cleavage. Importantly, it was shown that the dual tracrRNA:crRNA can be engineered as a single guide RNA (sgRNA) that retains two critical features: a 20 nucleotide sequence that determines the target site and a double-stranded structure that binds to Cas9. This created a simple two-component system in which changes to the 20 nucleotide guide sequence can be used to target CRISPR-Cas9 to virtually any DNA sequence of interest [3, 6].

Gene function can be disrupted by CRISPR-Cas9-mediated induction of double-strand breaks followed by nonhomologous end joining repair or gene replacement by homology-directed repair. Thus, we can now knock out any gene of interest in a wide variety of cells and organisms from all three domains of life [3]. However, the applications of CRISPR technology extend far beyond gene disruption: it also allows for specific changes of individual bases [7] or insertion of sequences encoding fluorescent proteins or epitope tags to study cellular protein functions without overexpression [5]. Another powerful application is the possibility to fine-tune gene function by CRISPR-Cas. Endonuclease-dead forms of Cas9 (dCas9) can be fused with transcriptional activation or repression effectors to control gene expression. Moreover, CRISPR-Cas9 allows for epigenetic alterations. Methyltransferases and acetyltransferases have been combined with dCas9 to modify gene regulation in a more physiological fashion [4]. CRISPR-Cas9 can be multiplexed to edit multiple genes at the same time and it has been used for genome-wide high-throughput screenings [9]. The versatility of CRISPR-mediated gene-editing makes it an ideal tool for physiological research, a field that studies biological systems at all levels of complexity.

CRISPR-Cas9 has inspired countless applications in basic science, agriculture, and medicine. Despite its tremendous translational potential, it should not be forgotten though that this powerful technology has emanated from curiosity-driven basic research. Remarkably, only eight years after its inception, clinical trials are underway to test whether CRISPR-Cas9 may be used to treat inherited diseases such as β-thalassemia or sickle cell disease [2]. For safe and effective clinical use, genome editing needs to be accurate, efficient, and deliverable to the desired cells or tissues. Scientists around the world are working tirelessly to achieve these goals and to advance the ever-expanding repertoire of genome editing technologies [2]. We are witnessing a biotechnological revolution.
